# Case Report of Orthopedic Screw Migration Presenting as Abdominal Pain in the Emergency Department

**DOI:** 10.5811/cpcem.50761

**Published:** 2026-10-06

**Authors:** Eric Jones, Marium Khan, Emmelyn J. Samones, Paul Brian Savino

**Affiliations:** * Loma Linda University Medical Center, Department of Emergency Medicine, Loma Linda, California; † Loma Linda University Health School of Medicine, Loma Linda, California

**Keywords:** abdominal pain, hip fracture, open reduction and internal fixation (ORIF), orthopedic screw migration, case report

## Abstract

**Introduction:**

Hip fractures are a concern in older adults, with incidence expected to rise as the population ages. Despite advances in surgical technique and hardware design, complications remain, influenced by patient factors and surgical choices. Rarely, hardware migration into the pelvis occurs, which poses serious risks. In the emergency setting, abdominal pain is a common complaint, and when evaluating patients with complex histories or prior orthopedic surgeries, clinicians must maintain a broad differential. This case details the discovery of a medially displaced hip screw into the pelvis as the cause of abdominal pain in a patient in the emergency department (ED).

**Case Report:**

A 44-year-old female with end stage renal disease on hemodialysis, anemia, and osteoporosis presented to the ED with sudden-onset, bilateral lower quadrant abdominal pain, vomiting, and diarrhea. The patient reported undergoing left hip open reduction and internal fixation seven months prior. Imaging of the abdomen noted medial displacement of the cephalomedullary nail through the left acetabulum into the pelvis with associated extraperitoneal hematoma. She underwent hardware removal and exploratory laparotomy without bowel injury.

**Conclusion:**

Orthopedic screw migration is a rare but serious complication, with limited documentation in the literature. Risk increases in patients with poor bone quality and healing, often due to chronic conditions. In patients with multiple comorbidities, careful history and a thorough exam when presenting to the ED are critical to identifying complications. Strategic use of imaging and clinical judgement can help emergency physicians refine their differential diagnosis.

## INTRODUCTION

Hip fractures remain a major public health concern and frequent presenting chief complaint in the emergency department (ED). In 2019, there were approximately 30,000 hip fracture-related ED visits in the United States among adults ≥ 65 years, a number that is anticipated to rise significantly with the continued aging of the population.^1^ Despite advances in surgical techniques and hardware design, complications remain common and often multifactorial in origin, with patient age, comorbidities, and procedural complications leading patients to return to the ED.^2^

Hardware migration following open reduction and internal fixation of the hip is a recognized complication; however, intrapelvic migration causing vascular injury or hematoma formation is rare and poses a significant diagnostic challenge. These patients often present with nonspecific abdominal or pelvic pain that mimics common conditions such as gastroenteritis, appendicitis, or urinary tract infection, and may not associate their remote surgical history with their current symptoms. Delayed recognition can result in life-threatening hemorrhage.

In this case report, we present a rare instance of abdominal pain initially attributed to food poisoning but found to be secondary to hardware failure involving medial displacement of a hip screw from an open reduction and internal fixation causing an intrapelvic hematoma. This case highlights the importance of maintaining a high index of suspicion and strong diagnostic vigilance when evaluating potential postoperative complications in the ED.

## CASE REPORT

A 44-year-old female with a past medical history significant for end-stage renal disease on hemodialysis with anemia and osteoporosis presented to the ED with sudden onset of bilateral lower quadrant abdominal pain for two hours associated with nonbloody vomiting and diarrhea. The patient stated that the pain began suddenly while she was driving and initially attributed the pain to a burrito she had eaten the night before. Her past surgical history included a surgical repair of a left hip fracture seven months prior, complicated by continued pain and need for a wheelchair since the procedure.

Initial vital signs were as follows: temperature, 99.1 °F; pulse, 82 beats per minute; blood pressure, 169/94 mm Hg; respiratory rate, 16 breaths per minute; and oxygen saturation, 94% on room air. On physical examination, the patient was found to be in distress. The abdominal exam revealed severe tenderness to palpation of the bilateral lower quadrants with noted distension, but no rebound. The upper abdomen was noted to be soft and nontender to palpation. She was also found to have significant tenderness to palpation of the left hip with severely limited range of motion secondary to pain. The patient was unable to bear weight due to severe bilateral hip pain, which was worse on the left compared to the right. Lab results were notable for a hemoglobin of 8.7 g/dL (reference range, 10.5–15 g/dL) and alkaline phosphatase of 1,196 U/L (37–132 U/L). Elevated alkaline phosphatase has been shown to be associated with both subacute fracture and bone remodeling after injury, which we suspected was the case here.^3^

Radiograph of the left hip was obtained due to left hip exam findings, which revealed postsurgical changes of open reduction and internal fixation with displacement of a cephalomedullary nail through the left acetabulum found to terminate within the pelvis (Image 1). Computed tomography (CT) of the abdomen and pelvis with contrast revealed similar findings with pelvis lesion consistent with an extraperitoneal hematoma. Orthopedic surgery and general surgery were consulted and recommended CT angiography (CTA) of the abdomen and pelvis to rule out arterial injury with ongoing bleeding. The CTA confirmed displacement of the cephalomedullary nail and associated pelvis lesion consistent with an extraperitoneal hematoma and possible venous bleed without arterial extravasation (Image 2).

*CPC-EM Capsule*

What do we already know about this clinical entity?

*Patients presenting to the emergency department with abdominal pain have a broad differential diagnosis.*

What makes this presentation of disease reportable?

*This patient presented with a rare case of an orthopedic hip screw that had migrated into the pelvis as the source of abdominal pain.*

What is the major learning point?

*A thorough history and physical exam, combined with a broad differential, can uncover rare diagnoses.*

How might this improve emergency medicine practice?

*It is important for emergency physicians to consider extra-abdominal sources as a potential cause of abdominal pain, especially in patients with comorbidities.*

The patient was administered hydromorphone and morphine for acute pain relief, as well as trimethobenzamide for nausea. She was admitted for surgical repair to both general and orthopedic surgery, where removal of hardware and exploratory laparotomy with abdominal washout was performed. She was noted to have poor bone quality with associated lack of fixation of prior fracture and no presence of bowel injury. The patient’s hospital course was complicated by a right femoral neck stress fracture discovered on day 10 and periprosthetic right femur fracture identified on day 19, requiring additional revisions. She was discharged home after a 23-day hospitalization with orthopedic surgery follow-up.

## DISCUSSION

Orthopedic screw migration following hip fracture fixation is a rare but life-threatening complication that poses diagnostic challenges in the ED. While medial screw migration is not uncommon and has been described in the literature, intrapelvic migration of the hardware resulting in vascular injury is rare. Its clinical presentation is often nonspecific and may mimic common gastrointestinal and gynecologic conditions.^4^,^5^

This case emphasizes several critical teaching points for emergency physicians. Any patient with a history of hip/pelvic hardware presenting with acute or acute-on-chronic abdominal or pelvic pain should prompt consideration of hardware-related complications in the differential diagnosis, regardless of how benign the presentation appears, or the time elapsed since surgery. Hardware migration may present with vague, nonspecific symptoms and normal vital signs due to a slowly progressive hematoma or hardware tamponade. Hardware migration may present along a clinical spectrum before catastrophic intrapelvic displacement occurs. Earlier signs include chronic hip or groin pain, decreased range of motion, pain with weight-bearing, or limb-length discrepancy, which are symptoms that may prompt repeat imaging and orthopedic referral before acute decompensation.^6^ Furthermore, this patient’s wheelchair dependence following open reduction and internal fixation, along with risk factors such as female sex and chronic kidney disease contributing to poor bone quality, warranted further evaluation of hardware placement.

Contrast-enhanced CT of the abdomen and pelvis is the diagnostic test of choice in a stable patient with acute-onset abdominal pain and history of pelvic hardware placement, as it allows for precise localization of possible complication/hardware migration, identification of contrast extravasation indicating arterial injury, and estimation of hematoma volume.^7^ While artifact secondary to hardware is a consideration, CT still provides critical diagnostic information regarding major pathology.^8^

Once intrapelvic hardware migration has been identified, ED management focuses on stabilization and preparation for definitive surgical intervention. Patients should be kept nonweight-bearing and immobilized to prevent further hardware displacement or vascular injury. In patients with signs of hemorrhagic shock, resuscitation should be initiated immediately with consideration of activation of a massive transfusion protocol for persistent shock, as delays are associated with increased mortality.^9^ If imaging demonstrates hardware proximity to bowel, free fluid, or free air concerning for perforation, empiric broad-spectrum antibiotics with Gram-negative and anaerobic coverage should be initiated.^10^

Management requires immediate multidisciplinary consultation with orthopedic surgery, trauma surgery, vascular surgery, and interventional radiology. Given the complexity of these cases and the need for multiple surgical subspecialties, definitive management typically requires transfer to a tertiary or quaternary care center. In this case, the combination of poor bone quality and intrapelvic migration necessitated explorative laparotomy for safe hardware extraction. Persistent or worsening pain after open reduction and internal fixation, particularly in patients who remain nonambulatory beyond expected recovery timelines, should raise concern for potential hardware complications in the ED.

## CONCLUSION

Migration of orthopedic hardware should be considered in ED patients with a history of prior fixation who present with new or persistent pain. In this case, the patient’s acute abdominal pain after eating a burrito—a seemingly benign trigger—could have led to diagnostic anchoring on a gastrointestinal cause despite the history of open reduction and internal fixation. Given the variable presentations of hardware migration, careful clinical judgment, thorough physical examination, and detailed history-taking remain essential especially when presenting to the ED. This case reinforces that emergency physicians must obtain a detailed surgical history in all patients with abdominal or pelvic pain and maintain a low threshold for contrast-enhanced CT imaging when prior hardware is present. Early recognition and prompt multidisciplinary involvement are critical to preventing the morbidity and mortality associated with this rare but serious complication.

## Figures and Tables

**Image 1:**
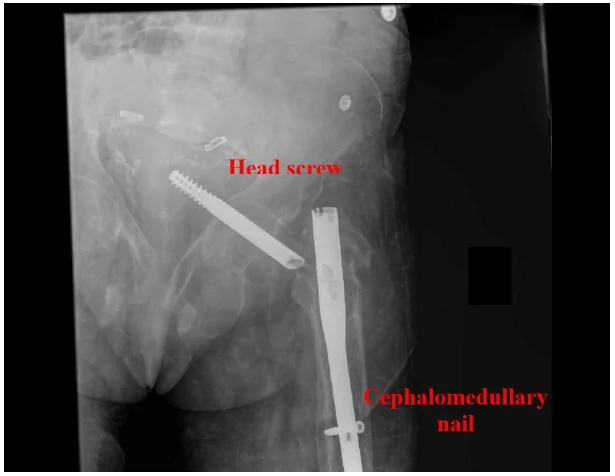
Anteroposterior radiograph of the left hip revealed postsurgical changes of open reduction internal fixation with displacement of cephalomedullary nail through the left acetabulum into the pelvis.

**Image 2:**
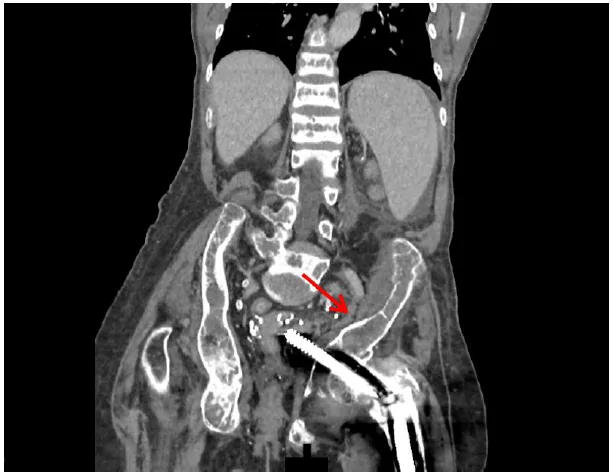
A coronal computed tomography angiogram of the abdomen and pelvis revealed postsurgical changes of left hip open reduction internal fixation with displacement of cephalomedullary nail through the left acetabulum into the pelvis with associated mixed density lesion suggestive of an extraperitoneal hematoma (arrow).
